# Reducing ageism combining ageing education with clinical practice: A prospective cohort study in health sciences students

**DOI:** 10.1002/nop2.1643

**Published:** 2023-02-20

**Authors:** Batirtze San‐Martín‐Gamboa, Idoia Zarrazquin, Ainhoa Fernandez‐Atutxa, Silvia Cepeda‐Miguel, Borja Doncel‐García, Idoia Imaz‐Aramburu, Amaia Irazusta, Ana B. Fraile‐Bermúdez

**Affiliations:** ^1^ Department of Nursing I, Faculty of Medicine and Nursing University of the Basque Country Leioa Bizkaia Spain; ^2^ Department of Nursing II, Faculty of Medicine and Nursing University of the Basque Country Donostia Spain; ^3^ OSI Bilbao‐Basurto, Osakidetza Basque Health Service Bilbao Bizkaia Spain; ^4^ Biocruces, Bizkaia Health Research Institute Barakaldo Spain

**Keywords:** ageism, nursing students, prejudices, stereotypes

## Abstract

**Aim:**

To analyse the impact of an intervention combining ageing education with clinical practice in nursing homes on a nursing cohort's negative stereotypes and prejudices towards ageing.

**Design:**

A prospective cohort study was conducted in September 2019–October 2020 in a population of health sciences students (*n* = 222).

**Methods:**

Questionnaire of Negative Stereotypes towards Aging (CENVE) and Aging Semantic Differential (DSE) were used to examine negative stereotypes and prejudices towards ageing in the nursing cohort exposed to the ageing education and practice intervention compared to a medical cohort that received no intervention. Group‐by‐time interaction, controlled by sex and age, for the effect of the intervention on CENVE and DSE scores was determined by mixed‐design ANOVA.

**Results:**

The nursing cohort significantly reduced negative stereotypes and prejudices towards ageing when compared to the medical cohort in total (*F =* 26.926; *p* < 0.001), health factor (*F =* 16.812; *p* < 0.001), motivational and social factor (*F =* 11.266; *p* = 0.001), and character and personality factor (*F =* 19.202; *p* < 0.001) scores of CENVE scale and in DSE (*F =* 7.826; *p* = 0.006).

## 1 INTRODUCTION

The world's population is ageing rapidly, and the proportion of people aged ≥65 years is expected to increase from 9.3% in 2020 to 16.0% in 2050 (United Nations, 2020). Despite our growing ageing population and the many contributions made by older people, negative attitudes towards this population are still common (Abrams & Swift, 2012). Robert Buttler was the first to define and contextualize the term “ageism” and described it as a set of stereotypes (cognitive component), prejudices (affective component) and discriminations (behavioural component) against people because of their age, which are reflected through behaviours such as contempt, dislike and indifference and can place ageing adults in vulnerable situations (Butler, 1969).

The coronavirus disease 2019 (COVID‐19) pandemic has highlighted ageism in society (Bravo‐Segal & Villar, 2020). Although COVID‐19 affects all age groups, most deaths occur in older populations and in those with previous health conditions or frailty (Grasselli et al., 2020; Hewitt et al., 2020; Richardson et al., 2020). Thus, older people are at the forefront as a risk group and a vulnerable population, influencing how they are viewed. In this context, many studies have warned of the potential for increased ageism and discrimination against older people during the COVID‐19 pandemic (Ehni & Wahl, 2020; Fraser et al., 2020; García‐Soler et al., 2020). The World Health Organization's recent Global Report on Ageism (World Health Organization, 2021) states that combating ageism is one of the four action areas of the Decade of Healthy Ageing (2021–2030) (Dixon, 2021).

## 2 BACKGROUND

Studies have shown that health sciences students can possess negative stereotypes of old age (Duran‐Badillo et al., 2016; Rello et al., 2018; Sequeira & Silva Jiménez, 2016), although contradictory data exist (Leon et al., 2015; Özdemir & Bilgili, 2016). Of note, studies examining these stereotypes were cross‐sectional and only revealed the relationship between different variables at a particular time, making it difficult to perform more in‐depth analyses. Further, validated questionnaires were not always used, and different components of ageism were not consistently explored. When ageist attitudes appear in health professionals, they negatively affect the health of older people because such attitudes lead to discriminatory behaviours, including inadequate health communication with the patient and family, underdiagnosis, lack of treatment, exclusion from research and denial of access to health services and treatment (Chang et al., 2020; Franco et al., 2010; Kagan & Melendez‐Torres, 2015; Makris et al., 2015). It is therefore important to implement actions to reduce these negative stereotypes and prejudices in health science trainees.

Several studies have observed that education about ageing may be effective in changing attitudes towards ageing in health sciences students (Basran et al., 2012; Rodgers & Gilmour, 2011; Sarabia‐Cobo & Castanedo‐Pfeiffer, 2015; Wurtele & Maruyama, 2013), yet few studies have used control groups to assess the effects of such educational interventions (Leung et al., 2012; Lucchetti et al., 2017). Contact with older people is recommended to decrease negative attitudes towards ageing (Burnes et al., 2019; Sum et al., 2016). However, for health science students, contact with older people often occurs during clinical practice, and exposure is usually with frail older adults, which may increase the risk of developing ageist attitudes (Giles et al., 2002).

Therefore, our goal was to analyse the impact of combining ageing education with clinical practice in nursing homes on a nursing cohort's negative stereotypes and prejudices towards ageing and compare outcomes with a medical cohort that did not receive the educational intervention. We hypothesized that this strategy would reduce negative stereotypes and prejudices towards ageing.

### 2.1 Research question

Could an educational intervention involving ageing education and clinical practice in nursing homes influence on a nursing cohort's negative stereotypes and prejudices towards ageing?

## 3 THE STUDY

### 3.1 Design

A prospective cohort study was used for this study.

### 3.2 Participants

The study was conducted in September 2019–October 2020 at the Faculty of Medicine and Nursing, University of the Basque Country, Spain. For the nursing cohort, in September 2019 we recruited 132 nursing students at the beginning of their second year of the Nursing Degree program who took a course with content pertinent to the care of older people and who were conducting clinical practice in nursing homes for the first time. The medical cohort consisted of 128 medical students at the beginning of their second year of the Medical Degree program. These students were not presented with educational content relevant to the care of older people and did not conduct clinical practice during the academic year. The final sample consisted of 113 nursing students and 109 medical students who completed the questionnaires at the beginning and end of the study.

### 3.3 Procedure

The nursing cohort was exposed to an educational intervention consisting of instructional and clinical components during the 2019–2020 academic year. The instructional component was provided during the second year of the Nursing Degree program and included content pertinent to gerontology and the care of older people. The COVID‐19 pandemic led to home confinement of the Spanish population in March–April 2020, but University of the Basque Country students continued attending online, and the course ended in May 2020 (Legido‐Quigley et al., 2020). The clinical component was a four‐week clinical practice (35 hours per week) in nursing homes performed in the second year of the Nursing Degree program during November 2019–February 2020. The medical cohort did not study any subjects related to ageing and did not do any clinical practice in the current or previous academic year.

Nursing and medical students were invited to several information sessions, which introduced the objective of the project, resolved doubts and requested their collaboration. All students interested in participating voluntarily signed informed consent forms and completed the questionnaires.

### 3.4 Materials

We used an ad hoc self‐administered questionnaire and collected sociodemographic information, including age, sex, marital status, number of family members and parents' education level. The Questionnaire of Negative Stereotypes towards Aging (CENVE) was used to examine the cognitive dimension of ageism (how we think) (Blanca et al., 2005). This questionnaire consists of 15 statements regarding the characteristics of older persons, with responses ranging from 1 (strongly disagree) to 4 (strongly agree); therefore, possible scores range 15–60, with higher scores indicating higher negative stereotypes. This questionnaire explores three factors: health factor, motivational and social factor, and character and personality factor. CENVE shows good psychometric properties with a Cronbach's alpha of 0.89 and is the most widely used instrument in Spanish‐speaking countries (Menéndez Álvarez‐Dardet et al., 2016; Rosell et al., 2020).

The Aging Semantic Differential (DSE) was used to examine prejudices towards ageing [i.e., affective dimension of ageism (how we feel)] and has an optimal level of reliability and high item correlation with a Cronbach's alpha of 0.91 (Villar Posada, 1997). This questionnaire includes 18 pairs of opposing adjectives that can be used to describe the ageing process. Each word pair is rated on a 7‐point scale, possible scores range 18–126 and lower scores reflect more negative prejudices.

### 3.5 Analysis

Statistical analysis was performed using the IBM SPSS Statistics v.26 statistical software package (SPSS Inc., Chicago, IL). Quantitative data were expressed as means (*M*) with standard deviations (*SD*) and categorical data as frequency counts (*n*) and percentages (%). Quantitative variables were checked for normality of distribution using the Kormogorov–Smirnov test. Differences of baseline characteristics between nursing and medical cohorts were compared by the independent student *t*‐test for quantitative variables and with the *chi*‐squared test for categorical variables. Effect sizes for those tests were calculated by Cohen's *d* and Cramer's *V* respectively. Differences in the evolution of CENVE and DSE scores between both groups were determined by mixed‐design ANOVA. Group‐by‐time effect controlled by sex and age was calculated. Effect size was determined by partial *eta*‐squared. *p‐*value <0.05 was considered statistically significant.

## 4 RESULTS

Sociodemographic characteristics of the final sample (*n* = 222) are shown in Table 1. Significant differences existed been nursing and medical cohorts. The nursing cohort was older (*t* = 3504, *p* = 0.001) and included more women (*Χ* ^*2*^ = 6.453, *p* = 0.011). The nursing cohort also had fewer family members (*t* = −2.376, *p* = 0.019) and significantly different education levels of both parents (*Χ* ^*2*^ = 16.563, *p* = 0.001 and *Χ* ^*2*^ = 18.697, *p* < 0.001, respectively). However, no differences were observed in baseline CENVE total score (*t* = −0.060, *p* = 0.990) and DSE score (*t* = 1.514, *p* = 0.167).

**TABLE 1:** Baseline characteristics of the study population by group.

Categorical variables	Nursing cohort, *n* = 113	Medical cohort, *n* = 109	Cramer´s *V*	*Χ* ^2^	df	*p*‐Value
Sex, *n* (*%*)						
Women	95 (84.1)	76 (69.7)	0.170	6.453	1	0.011
Men	18 (15.9)	33 (30.3)				
Marital status, *n* (*%*)						
Married	4 (3.5)	2 (1.8)	0.164	5.831	3	0.176
Single	88 (77.9)	98 (89.9)				
Separated/divorced	1 (0.9)	0 (0)				
Other	16 (14.2)	7 (6.4)				
No answer/don´t know	4 (3.5)	2 (1.8)				
Mother education level, *n* (*%*)						
No studies	1 (0.9)	1 (0.9)	0.274	16.563	3	0.001
Primary	18 (15.9)	7 (6.4)				
Secondary	39 (34.5)	21 (19.3)				
Higher education	52 (46.0)	80 (73.4)				
No answer/don´t know	3 (2.7)	0 (0)				
Father education level, *n* (*%*)						
Primary	25 (22.1)	6 (5.5)	0.294	18.697	2	<0.001
Secondary	45 (39.8)	38 (34.9)				
Higher education	38 (33,6)	64 (58.7)				
No answer/don´t know	5 (4.4)	1 (0.9)				

Quantitative variables	Nursing cohort *n* = 113	Medical cohort *n* = 109	Cohen´s *d*	*t*	df	*p*‐Value
Age, *M* (*SD*)	20.6 (4.49)	19.1 (0.96)	0.473	3.504	218	0.001
Family members, *M* (*SD*)	3.8 (0.77)	4.1 (0.77)	‐0.324	‐2.376	213	0.019
CENVE *M* (*SD*)						
Total score pre‐intervention	32.3 (5.42)	32.3 (5.42)	‐0.008	‐0.060	221	0.990
Health factor	10.5 (2.33)	10.7 (2.42)	‐0.063	‐0.474	221	0.636
Motivational and social factor	10.7 (2.32)	10.8 (2.24)	‐0.044	‐0.331	221	0.741
Character and personality factor	11.0 (2.39)	10.8 (2.37)	0.088	0.653	221	0.514
DSE *M* (*SD*)						
Total score pre‐intervention	74.1 (11.50)	71.8 (13.69)	‐0.016	1.514	220	0.167

Abbreviations: CENVE, Questionnaire of Negative Stereotypes about Aging; df, degree freedom; DSE, Questionnaire Semantic Differential of Aging; *M*, mean; *n*, frequency; *SD*, standard deviation; *t*, student´s *t*‐statistic; *Χ* ^*2*^, Chi‐ square value.

When analysing the effect of the intervention on negative stereotypes and prejudices towards ageing (Table 2), CENVE total score of the nursing cohort before intervention (*M* = 32.3, *SD* = 5.4) was significantly higher than after intervention (*M* = 26.5, *SD* = 6.0), indicating a statistically significant decrease in negative stereotypes towards ageing (*F* = 111.52, *p* < 0.001). Significant decreases were also observed in CENVE factors in the nursing cohort: health factor (*F* = 62.524, *p* < 0.001), motivational and social factor (*F* = 60.425; *p* < 0.001), and character and personality factor (*F* = 77.270, *p* < 0.001). In addition, DSE score before intervention (*M* = 59.8, *SD* = 31.1) was significantly lower than after intervention (*M* = 81.1, *SD* = 13.1), indicating a statistically significant decrease in prejudices towards ageing (*F* = 36.98, *p* < 0.001). There were no statistically significant differences in before‐ and after‐intervention CENVE (*F* = 0.01, *p* = 0.917) and DSE (*F* = 0.06, *p* = 0.601) scores in the medical cohort. The mixed ANOVA also revealed that nursing cohort improved more than medical cohort when analysing group‐by‐time interaction for CENVE total score (*F =* 26.926, *p* < 0.001) and all the CENVE factors; health factor (*F =* 16.812, *p* < 0.001), motivational and social factor (*F =* 11.266, *p* = 0.001), and CENVE character and personality factor (*F =* 19.202, *p* < 0.001). DSE score showed a higher improvement in nursing cohort comparing to medical cohort (*F =* 7.826, *p* = 0.006).

**TABLE 2:** Evolution of CENVE and DSE scores of nursing and medical cohorts.

			Nursing cohort, *n* = 113					Medical cohort, *n* = 109							Group‐by‐time effect				
	Pre‐test	Post‐test	Type III sum of squares	df	F	Within group *p‐*Value	Partial *eta‐*square	Pre‐test	Post‐test	Type III sum of squares	df	F	Within group *p‐* Value	Partial *eta*‐square	Type III sum of squares	df	F	p‐value	Partial *eta*‐square
Variable	*M* (*SD*)	*M* (*SD*)						*M* (*SD*)	*M* (*SD*)										
CENVE																			
Total score	32.3 (5.4)	26.5 (6.0)	1857.98	1	111.518	< 0.001	0.499	32.3 (5.4)	32.4 (5.8)	0.11	1	0.008	0.931	< 0.001	418.17	1	26.926	<0.001	0.111
Health factor	10.5 (2.3)	8.7 (2.4)	193.30	1	62.524	< 0.001	0.358	10.7 (2.4)	10.7 (2.5)	0.04	1	0.013	0.908	< 0.001	50.24	1	16.812	<0.001	0.073
Motivational and social factor	10.7 (2.3)	9.0 (2.3)	173.47	1	60.425	< 0.001	0.350	10.8 (2.2)	10.7 (2.5)	1.02	1	0.361	0.549	0.003	31.92	1	11.266	0.001	0.050
Character and Personality factor	11.0 (2.4)	8.9 (2.4)	256.99	1	77.270	< 0.001	0.408	10.8 (2.4)	11.1 (2.5)	1.82	1	0.611	0.436	0.006	60.43	1	19.202	<0.001	0.082
DSE																			
Total score	59.8 (31.1)	81.1 (13.1)	2685.14	1	36.985	< 0.001	0.248	71.6 (13.8)	71.4 (13.0)	4.13	1	0.057	0.811	< 0.001	557.00	1	7.826	0.006	0.035

*Note*: Group‐by‐time effect was controlled by sex and age.

Abbreviations: CENVE, Questionnaire of Negative Stereotypes about Aging; df, degree freedom; DSE, Questionnaire Semantic Differential of Aging; *F,* mixed ANOVA statistics; *M*, mean; *SD*, standard deviation.

Regarding covariates, there was no significant age‐by‐time interaction. However, sex‐by‐time interaction was significant for CENVE health factor (*F* = 4.901, *p* = 0.028), motivational and social factor (*F* = 4.344, *p* = 0.038) and total score (*F =* 7.035*, p = 0*.009) as women showed a greater improvement than men did.

## 5 DISCUSSION

We aimed to determine the impact of combining ageing education with clinical practice in nursing homes on a nursing cohort's negative stereotypes and prejudices towards ageing and compared outcomes with a medical cohort. Our results support the hypothesis that combining didactic education and clinical practice with older individuals significantly reduces negative stereotypes and prejudices about ageing in nursing students.

Several studies have conducted educational interventions to improve attitudes of health science students towards older people (Basran et al., 2012; Hwang et al., 2013; Rodgers & Gilmour, 2011; Sarabia‐Cobo & Castanedo‐Pfeiffer, 2015; Wurtele & Maruyama, 2013). Various educational interventions in geriatrics have the potential to improve student skills, knowledge and attitudes in geriatric medicine (Tullo et al., 2010). Further, pedagogical interventions designed to increase knowledge of ageing also can improve attitudes towards older adults (Chonody, 2015). However, previous studies examining how such interventions impact negative stereotypes towards older individuals vary considerably in both the methodology and the questionnaires used. In addition, these studies had limitations, validated questionnaires were not always used, the obtained results were not always significant, and many studies lacked a control group.

Control groups have been used in few studies assessing the effects of educational interventions on changing attitudes towards ageing in health sciences students. In medical trainees, in comparison to a control group receiving no intervention, implementation of two educational strategies has shown different outcomes in terms of attitudes, empathy and knowledge that depend on the strategy used (Lucchetti et al., 2017). However, that study did not focus on negative stereotypes and prejudices towards ageing and only conducted an educational intervention.

In addition, exploration of the long‐term effect of a 10‐week service‐learning project, where one older adult and one student met for 1–2 h per week, on medical and nursing students' knowledge of ageing and attitudes towards older adults demonstrated increased knowledge and reduced negative attitudes immediately after intervention (Leung et al., 2012). However, 1 month after the intervention, all groups showed a decrease in positive attitudes towards older adults. Curiously, the nursing student intervention group showed a greater decrease than the nursing student control group with no intervention, suggesting that the knowledge and attitude gains are limited (Leung et al., 2012). In the current study, the intervention was conducted during September 2019–May 2020, and the second data collection took place 4–5 months after intervention, in September–October 2020. Therefore, combining ageing education with clinical practice in nursing homes may be a viable strategy to maintain improvements in attitudes over time.

Recent studies built on the Positive Education about Aging and Contact Experiences (PEACE) model (Levy, 2016) have found improvements in attitudes towards ageing in undergraduates through positive intergenerational contact (Lytle et al., 2021; Lytle & Levy, 2019), an approach that has been supported by other studies (Burnes et al., 2019; Sum et al., 2016), but these studies were not carried out in nursing students. Positive intergenerational contact may be an effective strategy because exposure to only sick and frail older adults may place health professionals at greater risk of developing age‐discriminatory attitudes (Giles et al., 2002). In addition, conducting clinical practices in different facilities has various effects on students' attitudes and behaviours towards older people (Hwang et al., 2013). In the current study, there was contact between students and older people through a four‐week clinical practice in nursing homes, but the clinical practice was complemented with a course on gerontology and educational content pertinent to the care of older people. Previous studies have concluded that ageing education is important (Willetts et al., 2017) and highlighted that curricula need to be carefully planned to break down negative perceptions (Dahlke et al., 2020; Leung et al., 2012), and perhaps “be delivered by expert gerontological nurses to inspire undergraduate nursing students to care for older adults” (Neville et al., 2013). A literature review concluded that “relevant preparation and support allows students to engage in enriched learning experiences, deliver quality care, and develop positive attitudes in caring for older people in their professional practice” (Koh, 2012). Education on ageing is important, but curricula must ensure that such education is effective in reducing ageism. Considering the lack of interventions that apply the methodology proposed in the current study, linking clinical practice with education about ageing may be key to decreasing both negative stereotypes and prejudices in health sciences students.

### 5.1 Limitations

One of the limitations of this study is that the COVID‐19 pandemic occurred during our study, in the 2019–2020 academic year. It is possible that the pandemic influenced negative stereotypes and prejudices of health sciences students at the University of the Basque Country. Several studies indicate that ageism has increased (Ehni & Wahl, 2020; Fraser et al., 2020), but others show that the pandemic has promoted intergenerational solidarity (Previtali et al., 2020; Vervaecke & Meisner, 2020). Thus, the improvement in our nursing cohort could be due to the pandemic; however, the medical cohort did not show any changes in attitudes towards ageing, indicating that the observed positive changes were associated with the intervention and not the pandemic. Another limitation was that the two groups had different sociodemographic characteristics. Therefore, the results were not entirely free of bias. However, the baseline levels of stereotypes and prejudices about ageing were similar in both groups.

This study has important strengths. To the best of our knowledge, this study is the first to analyse both negative stereotypes and prejudice towards ageing in health sciences students; that is, our study explores ageism from its cognitive and affective components using validated questionnaires. Our study has an innovative, longitudinal design, where the intervention consists of a combination of didactic education about ageing and clinical practice in nursing homes, as well as use of a control comparison group. Our findings highlight the importance of implementing actions aimed at reducing ageism by targeting health sciences student education and training. This could improve the quality of care provided to the ageing population.

### 5.2 Implications for nursing education

Considering that combating ageism is one of the four action areas of the Decade of Healthy Ageing (2021–2030), educators have the responsibility to implement and analyse specific actions to reduce ageism among nursing students to ensure high quality of care provided to the ageing. These students will be dedicated to care in their future professional lives, and to a great extent, this care will be directed towards older people. It is essential to implement actions to reduce ageism among nursing and health science students, because without intervention there is no improvement, and discriminatory behaviours towards older people may be maintained.

## 6 CONCLUSION

Analysing possible discrimination against older people and identifying effective interventions are of vital importance, especially in professions that work directly with ageing populations, such as health professionals. Our results suggest that an intervention combining ageing education with clinical practice in nursing homes can significantly reduce negative stereotypes and prejudices about ageing in nursing students. These observed cognitive and affective changes could lead to behavioural changes, which could improve the quality of care provided to older people. These results will benefit nursing students, future health professionals, older people and society and serve as a foundation for further research aimed at developing more inclusive interventions and education for both nursing and health sciences studies.

## AUTHOR CONTRIBUTIONS

**Batirtze San‐Martín‐Gamboa**: Conceptualization, Investigation, Writing – review & editing, Data curation. **Idoia Zarrazquin**: Conceptualization, Investigation, Writing – review & editing. **Ainhoa Fernández‐Atutxa**: Methodology, Software, Validation, Formal analysis. **Silvia Cepeda‐Miguel**: Investigation, Data curation, Writing – review & editing. **Borja Doncel‐García**: Conceptualization, Investigation, Writing – review & editing. **Idoia Imaz‐Aramburu**: Investigation, Resources. **Amaia Irazusta**: Conceptualization, Funding acquisition, Visualization. **Ana Belén Fraile‐Bermúdez**: Conceptualization, Project administration, Writing – original draft, Supervision.

## FUNDING INFORMATION

This research received no specific grant from any funding agency in the public, commercial or not‐for‐profit sectors.

## CONFLICT OF INTEREST STATEMENT

The authors report no conflict of interest.

## RESEARCH ETHICS COMMITTEE APPROVAL

The study was conducted in accordance with the Declaration of Helsinki and was approved by the Committee on Ethics in Research of the University of the Basque Country (Code M10/2019/143MR1).

## Data Availability

Data available on request due to privacy/ethical restrictions
